# Chronic Intermittent Sucrose Consumption Facilitates the Ability to Discriminate Opioid Receptor Blockade with Naltrexone in Rats

**DOI:** 10.3390/nu14050926

**Published:** 2022-02-22

**Authors:** David C. Jewett, Donisha S. N. K. Liyanagamage, Mark A. Vanden Avond, Molly A. B. Anderson, Kyleigh A. Twaroski, Morgan A. Marek, Kimberly F. James, Tapasya Pal, Anica Klockars, Pawel K. Olszewski, Allen S. Levine

**Affiliations:** 1 Department of Psychology, University of Wisconsin-Eau Claire, Eau Claire, WI 54701, USA; jewettd@uwec.edu (D.C.J.); mvandenavond@mcw.edu (M.A.V.A.); molly.a.b.anderson@dartmouth.edu (M.A.B.A.); kyleigh.twaroski@gmail.com (K.A.T.); morgan.marek1220@gmail.com (M.A.M.); kfjames@buffalo.edu (K.F.J.); 2 Faculty of Science and Engineering, University of Waikato, Hamilton 3240, New Zealand; donishaliyanagama87@gmail.com (D.S.N.K.L.); paltapasya23@gmail.com (T.P.); anica.klockars@waikato.ac.nz (A.K.); pawel.olszewski@waikato.ac.nz (P.K.O.); 3 Department of Integrative Biology and Physiology, Medical School, University of Minnesota, Minneapolis, MN 55414, USA; 4 Department of Food Science and Nutrition, University of Minnesota, St. Paul, MN 55108, USA

**Keywords:** sucrose, drug discrimination, naltrexone, c-Fos IR, opioid, brain

## Abstract

The opioid antagonist naltrexone (NTX) decreases intake of preferred diets in rats at very low doses relative to doses needed to decrease intake of “bland” laboratory chow. In the absence of an opioid agonist, NTX is not discriminable using operant techniques. In the current study, we found that rats given intermittent access to a 25% sucrose solution learned to discriminate between various naltrexone doses and saline. None of the rats given only water learned to discriminate between naltrexone and saline. When access to the sucrose solution was discontinued for 14 days, the rats lost the ability to discriminate between NTX and saline. We also studied the changes of c-Fos IR in selected brain regions in rats treated with saline versus NTX that were drinking water or 25% sucrose. An injection of NTX or saline resulted in a significant drug, diet, and interaction effect in various brain regions associated with feeding behavior, particularly the amygdala, accumbens, and hypothalamic sites. Thus, we found that ingestion of a sucrose solution results in the ability of rats to reliably discriminate naltrexone administration. In addition, sucrose and naltrexone altered c-Fos IR in an interactive fashion in brain regions known to be involved in ingestion behavior.

## 1. Introduction

It is well established that opioid circuits are involved in the regulation of food intake [1,2,3]. In laboratory animals, agonists of opioid receptors increase, and antagonists decrease food intake. Early studies suggested that opioid agonists selectively increased dietary fat consumption [4,5]. However, Gosnell [6] demonstrated that morphine increased intake of high-fat diets in rats that preferred such diets, and it produced a similar orexigenic effect in carbohydrate preferers given high-carbohydrate food. Our laboratory published a series of papers that support the latter finding [7,8,9,10].

There is a strong interaction between sugars and opioids in central regulation of feeding and drinking. Naloxone, an opioid antagonist, reduces imbibition of sucrose or saccharin solutions more effectively than intake of water even in sham feeding paradigms [11,12]. Naloxone can also reduce intake of sucrose solutions in sham-fed animals, suggesting that postabsorptive signaling is not necessary for a decrease in sucrose intake following naloxone administration [12]. Naloxone does not affect sucrose discrimination in rats trained to discriminate 10% sucrose from water [13]. Humans also continue to discriminate sucrose solutions after opioid receptor blockade [14]. Interest in the longer-acting opioid antagonist naltrexone (NTX) has increased, as this molecule has been clinically tested to decrease addictive behaviors (from alcohol drinking to gambling), and it has been a component of the recently approved anti-obesity medication Contrave (NTX + bupropion) as well as part of combination pharmacotherapies (with oxytocin) in hypothalamic obesity case studies [15,16,17,18]. In rats, NTX decreases intake of standard chow and a 32% sucrose solution more effectively than standard chow alone [19]. NTX is effective in decreasing intake of palatable high-fat, high-sugar chow [20,21].

However, opioid receptor antagonists decrease the positive hedonic effect of sugar, that is, pleasantness of sugar in both humans and non-human animals. When given a choice between a high-sugar diet and a high-starch diet, virtually all rats prefer the high sugar diet. Our laboratory demonstrated that chronic central infusion of naltrexone via a mini-osmotic pump inhibited the re-development of a preference for a high-sugar diet after a period of ingestion of only a less rewarding starch diet [22].

In addition to opioids affecting ingestion of sweet, palatable diets, sweet foods can affect opioid circuitry. For example, opioid gene expression in the arcuate nucleus is altered by ingestion of a high-fat, high-sugar diet [23]. Restricted feeding of this preferred diet feeding pair to intake of a bland chow resulted in a decrease in gene expression of opioids, a response similar to that seen with energy deprivation [24]. Some have suggested that sugar ingestion can lead to addictive behaviors similar to those seen with opiates. Colantuoni and colleagues [25] exposed rats to an extended period of intermittent access to sucrose. They then injected these rats with naloxone to evaluate whether chronic sucrose ingestion led to opioid-like withdrawal. They reported withdrawal symptoms, such as teeth chattering, forepaw tremor, and head shakes. Hoebel’s laboratory [26,27] also found that opioid blockade following sugar consumption in rats resulted in a ratio of acetylcholine to dopamine in the CNS, which resembled that seen with morphine withdrawal. Pomonis et al. [28] studied the effect of three weeks of consumption of a 10% sucrose solution on naloxone-induced changes in c-Fos immunoreactivity (IR) in the brain of rats. The greatest effect was seen in the central nucleus of the amygdala (CEA). Naloxone injection in rats drinking water instead of sucrose increased c-Fos-IR in the CEA, and sucrose significantly enhanced this effect. Koob [29,30] noted that the amygdala, especially the CEA, has been implicated in opioid dependence, and negative aspects of opioid withdrawal are mediated largely via this site. Further evidence of sucrose affecting opioid circuits was demonstrated in a study by Jewett et al. They reported that chronic sucrose ingestion enhanced the ability of rats to discriminate doses of the opiate nalbuphine by three-fold [31].

A variety of reports indicate that rats cannot discriminate NTX from saline, except at extraordinarily high doses, in operant paradigms [32,33,34]. Animals can discriminate opioid antagonists when subjects are treated chronically with an opioid agonist [28]. However, in those previous studies, the animals had not been receiving a highly palatable, sugary food in an intermittent fashion. Based on the data discussed above, we hypothesized that chronic, intermittent sucrose consumption would increase opioid function. Naltrexone would produce a reduction in this sucrose-induced effect and allow rats to discriminate NTX from saline. In order to better understand neural changes associated with the ability to discriminate NTX in sucrose-given animals, we examined concurrent changes in c-Fos IR expression associated with the diet and drug treatment that paralleled the successful discrimination training regimen in a variety of brain sites known to be involved in the regulation of food intake. The c-Fos study was crucial not only from the standpoint of a different opioid antagonist used by us versus Pomonis but also because the animals’ exposure to sucrose was intermittent, thus generating a more robust appetitive response (likely driven by reward processes) than the unrestricted access to sugar utilized in the earlier report.

## 2. Materials and Methods

### 2.1. Animals

Male Sprague–Dawley rats (Harlan, Madison, WI, USA, approximately 12 weeks old at the beginning of the study) were housed individually in standard polycarbonate cages under a 12:12 light:dark cycle (lights on at 6:00 a.m. in all discrimination studies) in a temperature-controlled (22 °C) laboratory animal facility. Standard rodent chow (Harlan Teklad, Madison, WI, USA) and tap water were available ad libitum unless noted. Animals were maintained in accordance with the National Institute of Health Guide for the Care and Use of Laboratory Animals. The University of Wisconsin-Eau Claire Institutional Animal Care and Use Committee and the University of Waikato Animal Ethics Committee approved all experimental manipulations described in this project.

### 2.2. Chemicals

Naltrexone (Sigma Chemical Co., St. Louis, MO, USA) was dissolved in 0.9% saline and administered SC in a volume of 1 mL/kg. Commercially available sucrose was dissolved in tap water.

### 2.3. Operant Discrimination Studies

#### 2.3.1. Establishing 3.2 mg/kg Naltrexone as a Discriminative Stimulus in Rats Given Chronic, Intermittent Sucrose Access

Rats were trained to lever press in standard operant chambers (Med-Associates, St. Albans, VT, USA). These chambers were placed in ventilated, sound-attenuating cubicles equipped with fans. Forty-five milligrams of F#0021 food pellets (Bio-Serve, Frenchtown, NJ, USA) were used to reinforce lever pressing and delivered by a dispenser into a food trough located between the two response levers. The operant chamber was illuminated by a house light in the back panel during experimental sessions. Experimental contingencies and data acquisition were done via Med Associates software (St. Albens, VT, USA) and a computer located in a separate room.

Sixteen rats were initially food restricted to reach ~85% of their free-feeding body weight and trained to lever press. At first, a single-lever press (fixed ratio 1, FR 1) was reinforced with a 45-mg pellet. The number or responses required to earn food gradually increased until 15 lever presses (FR 15) were required for each pellet delivery. When pressing occurred reliably at both levers, animals regained unrestricted access to chow. At 6 p.m. daily (the start of the dark phase of the LD cycle), rats received a bottle containing 25% (*n* = 6) or 32% (*n* = 10) sucrose or water (*n* = 4) at 6 p.m. At 6 a.m., all rats were given a bottle containing water. This pattern of intermittent sucrose access continued throughout the study unless otherwise indicated.

Fourteen days later, discrimination training began. At the onset of the dark phase (6 p.m.), rats were given sucrose or water as described above. One hour later, rats received a subcutaneous injection of 3.2 mg/kg naltrexone (NTX) or saline. After the injection (NTX or saline), rats were placed into the operant chamber 15 min before discrimination training. During training sessions, the house light was illuminated, and 15 correct lever presses (FR 15; left lever presses following NTX administration and right lever presses following saline administration) were reinforced with the 45-mg pellet delivery. Incorrect lever presses (right lever presses following NTX injection and left lever presses following saline administration) were punished with 8 s of darkness under a FR 15 schedule. Training continued until 5 reinforcers were earned or 15 min elapsed. After the training session, rats were returned to the home cage with access to supplemental laboratory rat chow and the sucrose solution. Discrimination training continued until the individual emitted at least 80% of condition-appropriate responses before delivery of the first reinforcer and for the whole training session during all training cycles for 8 (out of 10) consecutive daily sessions.

Acquisition data from different NTX training doses were compared using ANOVA followed by Tukey *t*-tests if the ANOVA revealed statistical significance. Differences were considered statistically significant for *p* ≤ 0.05.

#### 2.3.2. Discrimination Procedure to Assess Different Training Doses of NTX

Thirty-two experimentally naïve rats were trained to lever press as described above. Sucrose access (25% sucrose solution) and discrimination training were implemented as described above. Rats were divided into three groups. Rats were trained to discriminate between 1.0 mg/kg NTX and saline (*n* = 4), 0.32 mg/kg NTX and saline (*n* = 7), or 0.1 mg/kg NTX and saline (*n* = 21). Discrimination training continued until the discrimination criteria described in Section 2.3.1 were met. Acquisition data from different NTX training doses were compared with ANOVA followed by Tukey *t*-test. Differences were considered statistically significant for *p* ≤ 0.05.

#### 2.3.3. Generalization Tests I: Constructing Naltrexone Dose-Effect Functions

After subjects acquired the discrimination, generalization tests were conducted. Sucrose solution (25%) was given one hour before the test session. A dose of NTX or saline was injected 15 min prior to a test session. During generalization tests, the house light was illuminated, and 15 lever presses to either lever were reinforced with the 45-mg pellet delivery. The test session continued until 5 reinforcers were earned or 5 min elapsed. After the test, the subject was returned to the home cage and given supplemental feeding and access to 25% sucrose for the remainder of the dark cycle. To ensure discriminative performance remained reliable, appropriate performance for two consecutive training sessions (one preceded by NTX and one preceded by saline) were required between generalization tests.

For each training dose, generalization data from different naltrexone doses were compared with ANOVA followed by Dunnett’s test. Differences were considered significant for *p* ≤ 0.05. Rates of lever pressing following different NTX doses were compared with repeated measures ANOVA followed by Dunnett’s test. Differences were considered significant for *p* ≤ 0.05.

#### 2.3.4. Generalization Tests II: Effects of Acute Water Consumption on the Discriminative Stimulus Effects of Naltrexone

If sucrose consumption produced a short-term increase in opioid function, we would expect naltrexone’s ability to serve as a discriminative stimulus to be reduced if rats were given water 1 h before the test session. To test this possibility, a subset of rats from experiment 2.3.1 were given 1 h access to water at the beginning of the dark cycle (6 p.m.). Fifteen minutes before the experimental session, rats were injected with naltrexone (0.032–3.2 mg/kg) or saline. Generalization test sessions were conducted as described above. After the test sessions, rats were returned to their home cages and received access to a 25% sucrose solution until 6 a.m. Discrimination training resumed with sucrose access the next day. Another generalization test with a different NTX dose was given after the subject met the discrimination performance criteria for two consecutive training sessions (one preceded by NTX and the other by saline).

#### 2.3.5. Effects of Chronic Water Consumption on the Discriminative Stimulus Effects of Naltrexone

Rats from experiment 2.3.4 were then given 24-h access to water for 14 days. Discrimination training resumed. Water bottles were changed at 6 p.m. One hour later, rats were injected with the training dose of naltrexone or saline, and discrimination training sessions were described as above. After the test sessions, rats were returned to their home cages and received access to water until 6 a.m. At 6 a.m., the water bottle was replaced by another water bottle. This daily procedure continued for 11 days. After the 11 days, discrimination training was suspended. Rats were given 14 days of chronic, intermittent sucrose access. Following the 14 days of chronic, intermittent sucrose access, discrimination training resumed as indicated above (2.3.1).

### 2.4. c-Fos Studies: Effect of an NTX/Saline Injection on c-Fos Immunoreactivity in Rats Drinking Sucrose vs. Water Subjected to a Random Daily NTX/Saline Injection Regimen

Rats were subdivided into two cohorts. The control diet cohort was maintained on standard chow and tap water ad libitum. The palatable sucrose diet cohort was given every night access to a bottle containing 25% sucrose solution: freshly made sucrose was given at 6:00 p.m. and taken away at 6:00 a.m. Water was removed for the time of sucrose presentation and returned to the cages for the duration of the light phase of the LD cycle. In order to minimize the effects of excessive energy intake on body weight in the sucrose cohort, these animals were given 10 g of standard chow per day.

In order to mimic the length of the treatment in operant studies that led to NTX discrimination and familiarize the animals with the interoceptive effects of NTX versus saline injection, the exposure to the tastants and the injectants lasted for 10 weeks. During those weeks, the animals received a single daily injection of either saline or 1 mg/kg NTX (at 7:00 p.m.) according to the random schedule (half of the injections were saline and the other half NTX). On the final day, the animals were injected with either saline or NTX (7:00 p.m.). Chow and tap water were taken away from the cages right after the injection. An hour later, the animals were anesthetized with urethane (35% in isotonic NaCl) and perfused via the aorta with 50 mL saline (room temperature) followed by 400 mL of 4% paraformaldehyde (ice-cold; in 0.1 M phosphate buffer).

Brains were dissected and immersed for post-fixation in 4% paraformaldehyde at 4 °C. After 24 h, the brains were sliced with the vibratome (Leica, Germany; 60-μm coronal sections). Free-floating sections were used for c-Fos immunohistochemistry.

Sections were washed in 50 nM TBS (pH 7.4–7.6) four times and then treated in 3% H_2_O_2_ + 10% methanol (in TBS; 10 min). After four washes in TBS, they were incubated for 12 h in the polyclonal antibody against c-Fos (made in rabbit; 1:4000; Synaptic Systems, Australia; 4 °C). The tissue was then rinsed in TBS and incubated for 60 min in the goat-anti-rabbit secondary antibody (1:400; Vector Laboratories, Burlingame, CA, USA; room temperature). Afterwards, sections were washed in TBS four times and incubated for 60 min with the avidin–biotin peroxidase complex (ABC; 1:800; Elite Kit, Vector Laboratories, Burlingame, CA, USA). The incubations were done in the 0.25% gelatin + 0.5% Triton X-100 solution (in TBS). The peroxidase was visualized with 0.05% diaminobenzidine (DAB), 0.01% H_2_O_2_, and 0.3% nickel sulfate in TBS.

The stained sections were mounted onto gelatin-coated slides, air-dried for 24 h, dehydrated in alcohol for 20 min, soaked in xylene (Merck KGaA, Darmstadt, Germany), and embedded in Entellan (Merck KGaA, Darmstadt, Germany). Images were acquired via a camera attached to a light microscope (Nikon Eclipse 400). The number of Fos-immunoreactive nuclei per mm^2^ of tissue was counted bilaterally for each brain area with ImageJ Software. Site boundaries were determined with the aid of the Paxinos and Watson atlas on 2–4 sections per rat. Means and SEM were calculated and data compared with two-way ANOVA with drug and diet set as independent factors.

## 3. Results

**Figure 1:**
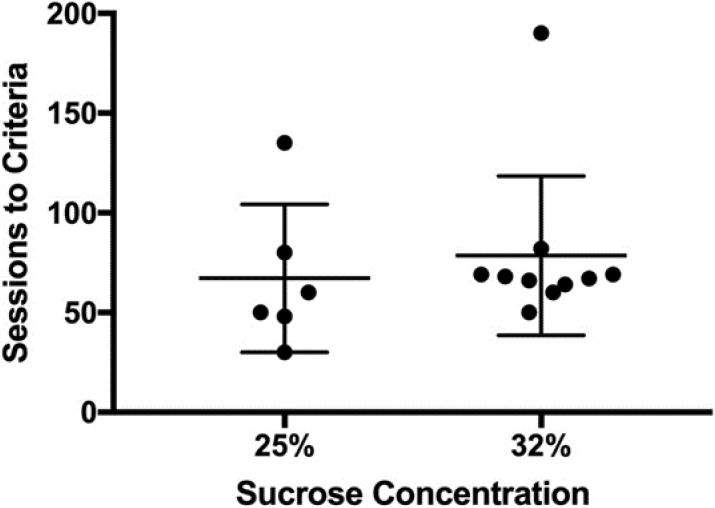
Effect of sucrose solution concentration on acquisition of the discrimination between 3.2 NTX and saline. Filled symbols represent individual subject sessions to discrimination criteria. The long horizontal line represents the mean sessions to criteria and the shorter horizonal line represents SEM. No significant differences between the concentrations were noted.

Rats with intermittent access to sucrose learned to discriminate between 3.2 mg/kg NTX and saline (Figure 1). Sucrose concentration had no significant impact on the discrimination acquisition. The sessions to discrimination criteria were not different between rats maintained under 25% sucrose access (M = 55, SEM = 15 sessions) and rats maintained under 32% sucrose (M = 68, SEM = 13 sessions), t_(14)_ = 0.56 *p* = 0.58.

**Figure 2:**
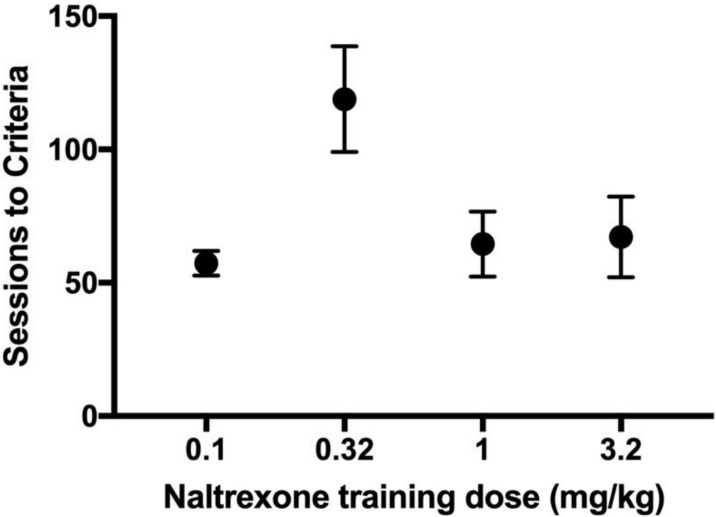
Effect of NTX training dose (0.01, 0.32, 1.0, and 3.2 mg/kg) on discrimination acquisition. Filled symbols represent mean sessions to criteria (+/− SEM) for each training dose. No significant differences among training doses were noted.

Rats with intermittent access to 25% sucrose reliably learned to discriminate between subcutaneous injections of NTX (0.32–3.2 mg/kg as training doses, Figure 2). The sessions to discrimination criteria were not different among rats trained with a training dose of 3.2 mg/kg (M = 68, SEM = 13 sessions), 1.0 mg/kg (M = 64.5, SEM = 12 sessions), or 0.32 mg/kg (M = 119, SEM = 20 sessions), F_(2,14)_ = 3.20, *p* = 0.07. In rats given 0.1 mg/kg naltrexone as the training dose, 7 of 21 subjects learned the discrimination, and training of the other 14 subjects was terminated after 81 sessions. No subjects maintained under chronic water-access conditions learned the discrimination within 76 training sessions.

**Figure 3:**
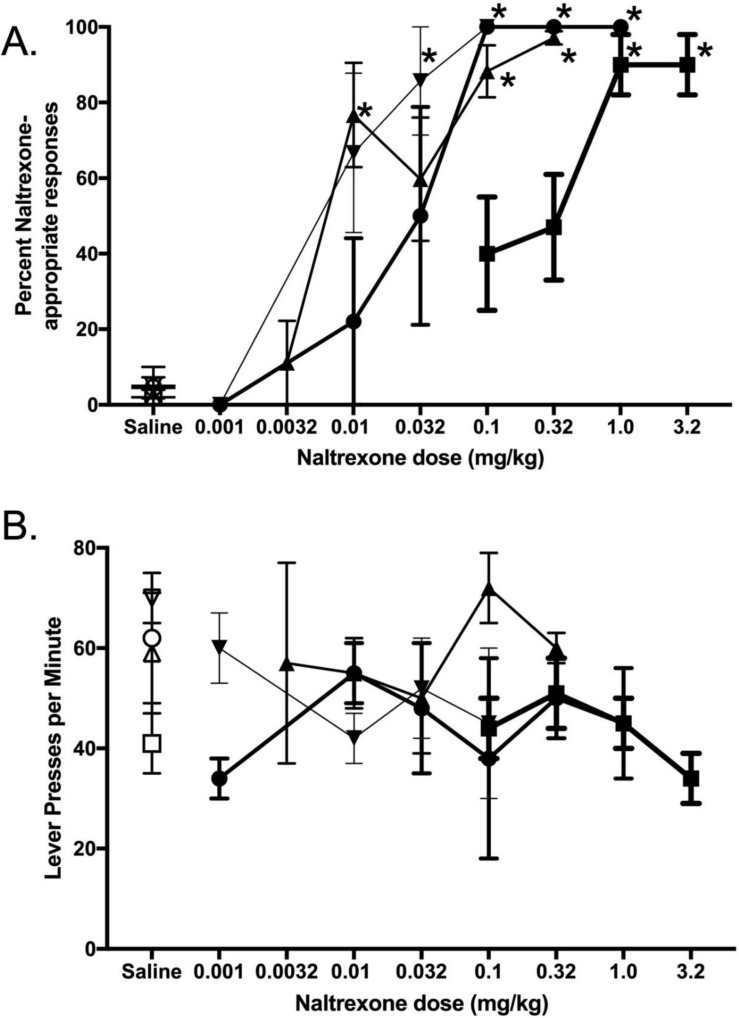
NTX dose-effect curves are depicted as a function of training dose (0.1 mg/kg NTX inverted triangle, 0.32 mg/kg NTX triangle, 1.0 mg/kg NTX circle, and 3.2 mg/kg NTX square). (**A**) NTX generalization curves as a function of naltrexone training dose. The percentage of responses associated with the NTX training dose is plotted on the ordinate. (**B**) Response rate (lever presses per minute) is plotted as a function of NTX dose. * = *p* < 0.05 (significantly different than saline control).

Naltrexone produced dose-dependent increases in NTX training dose-discriminative stimulus effects (Figure 3). Naltrexone’s potency to generalize to the training dose was inversely related to the training dose (smaller NTX doses generalized to 0.01 mg/kg, whereas larger NTX doses generalized to 3.2 mg/kg training dose). There was a main effect of naltrexone dose on the percentage of NTX-appropriate responses for each training dose, 0.1 mg/kg NTX F_(4,33)_ = 20.42, *p* < 0.001; 0.32 mg/kg NTX F_(6,46)_ = 6.09, *p* < 0.001; 1.0 mg/kg NTX F_(4,24)_ = 20.05, *p* < 0.001; 3.2 mg/kg NTX F_(4,51)_ = 11.56, *p* < 0.001. Response rates were not significantly affected by NTX (Figure 3B).

**Figure 4:**
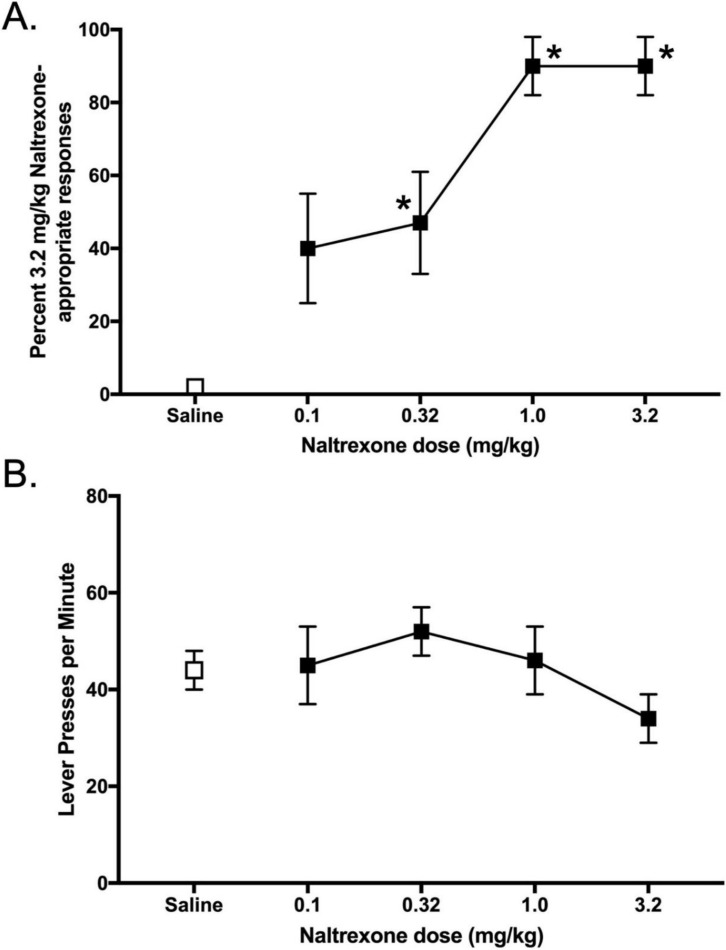

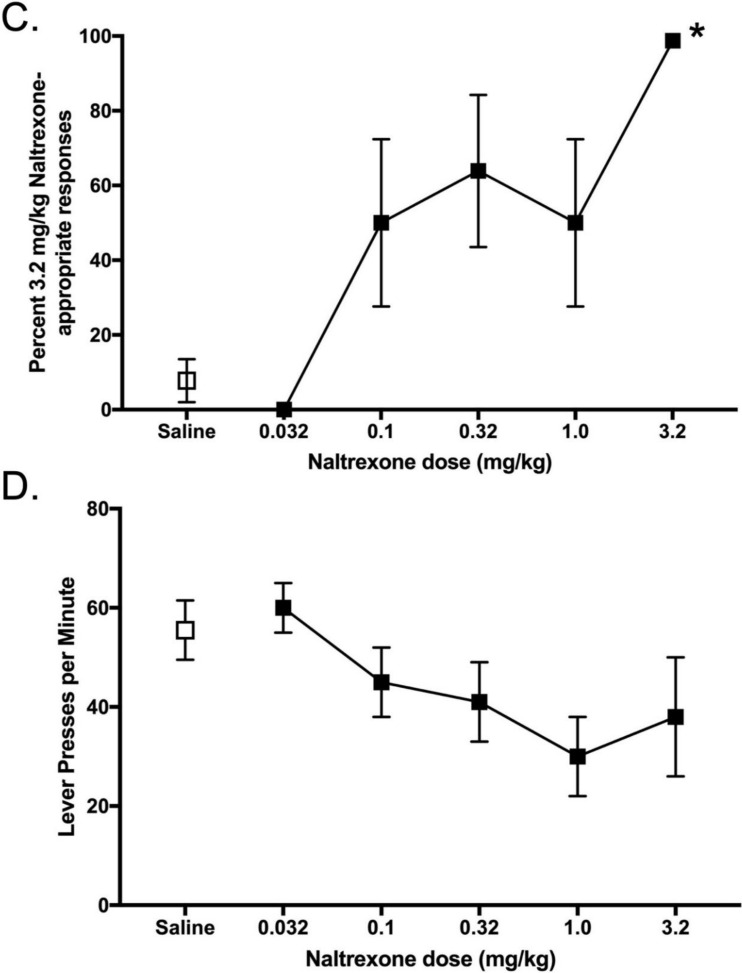
Effect of acute water substitution on NTX generalization functions (saline, open squares and NTX, filled squares) in rats trained to discriminate 3.2 mg/kg NTX from saline. (**A**) NTX generalization function and (**B**) responses rates in rats with chronic intermittent 25% sucrose solution given 1 h before the test. (**C**) NTX generalization function and (**D**) responses rates in rats with water given 1 h before the test. * = *p* < 0.05 (significantly different than saline control).

Naltrexone’s ability to serve as a discriminative stimulus was not altered when water was substituted for 25% sucrose 1 h before the injection of NTX (Figure 4). In rats maintained under intermittent sucrose access, 0.32 mg/kg NTX produced a half-maximal effect (Figure 4A), and response rates were not altered by NTX (Figure 4B). When water was given 1 h before the test session instead of 25% sucrose, the discriminative stimulus effects were not altered (Figure 4C). The dose to produce a half maximal effect was similar (~0.32 NTX), and most doses tested produced similar increases in NTX-appropriate responding. Response rates (Figure 4D) were also not significantly affected by NTX when water was substituted for 25% sucrose solution 1 h before the test session.

**Figure 5:**
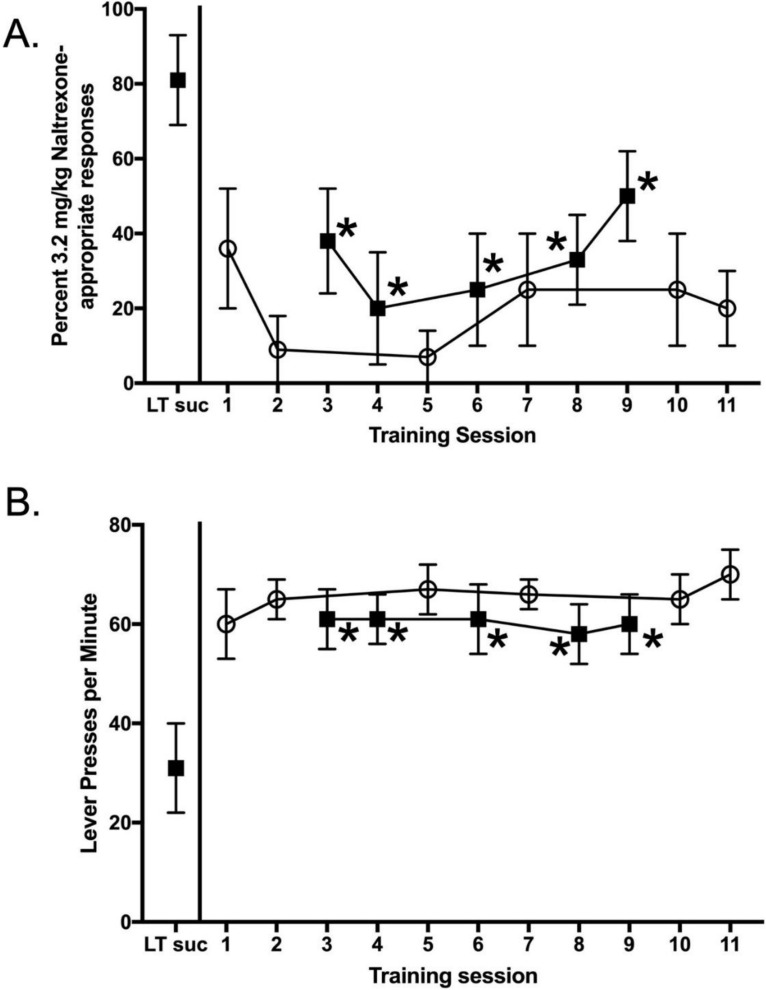
Effect of 14-day water access on the ability of NTX to serve as a discriminative stimulus. (**A**) NTX-appropriate responding and (**B**) rates of lever pressing for NTX (filled symbols) and saline (open symbols) are plotted as a function of training session. The point above LT suc represents data from the last 3.2 NTX training day before training was suspended, and water access began for 14 days. Training days 1–11 were conducted under water-access conditions. * = *p* < 0.05 (significantly different than the last NTX training day under chronic, intermittent sucrose conditions).

**Figure 6:**
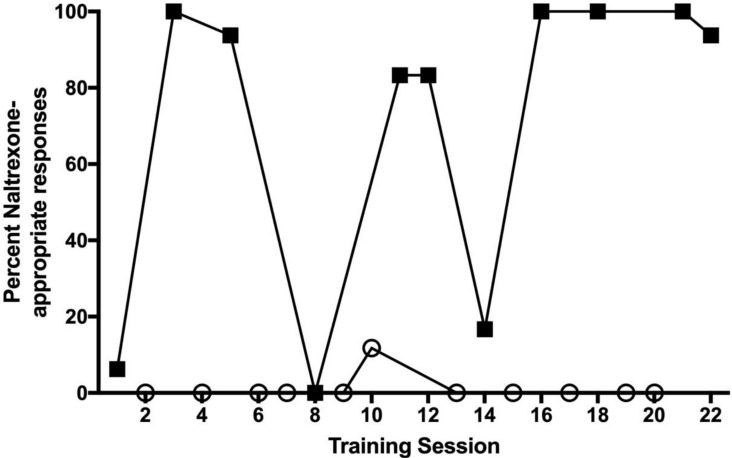
Reacquisition of NTX as a discriminative stimulus after 14 days sucrose access (25%). Data for NTX training sessions (filled squares) and saline training sessions (open circles) from a representative subject are shown. NTX-appropriate responding is plotted as a function of training session.

Rats maintained under chronic intermittent sucrose and trained to discriminate 3.2 mg/kg NTX from saline were given 14 days access to water and resumed discrimination training while maintained on water. Under chronic water access, the discriminative stimulus effects of NTX were significantly reduced (Figure 5A). F_(5,59)_ = 2.51, *p* = 0.041. Under these conditions, performances following during saline training sessions did not meet the discrimination performance criteria. Subjects lost the ability to discriminate between 3.2 mg/kg NTX and saline. Response rates were also significantly higher for NTX training days conducted under the chronic water conditions F_(5,59)_ = 3.00, *p* = 0.018 (Figure 5B). When access to 25% sucrose was reinstated for 14 days, rats readily relearned to discriminate between NTX and saline. Figure 6 shows the discrimination reacquisition data for a typical subject. As the training days increased, data tended to be at the top of the graph following NTX administration and at the bottom of the graph following saline administration, indicating the rat was reliably discriminating between 3.2 mg/kg NTX and saline.

In c-Fos studies, a two-way ANOVA analysis showed a significant effect of the drug in the PVN (*p* < 0.0001, F_(1,16_) = 168.6), SON (*p* < 0.0001, F(_1,16)_ = 50.26), ARC (*p* < 0.0001, F_(1,16)_ = 74.05), DMH (*p* = 0.0019, F_(1,16)_ = 13.69), VMH (*p* < 0.0001, F_(1,16)_ = 53.81), CEA (*p* < 0.0001, F_(1,16)_ = 773.9), BLA (*p* < 0.0001, F_(1,16_ =33.86), AcbS (*p* < 0.000, F_(1,16)_ = 36.06), and NTS (*p* = 0.0145, F_(1,16)_ = 7.514).

**Figure 7:**
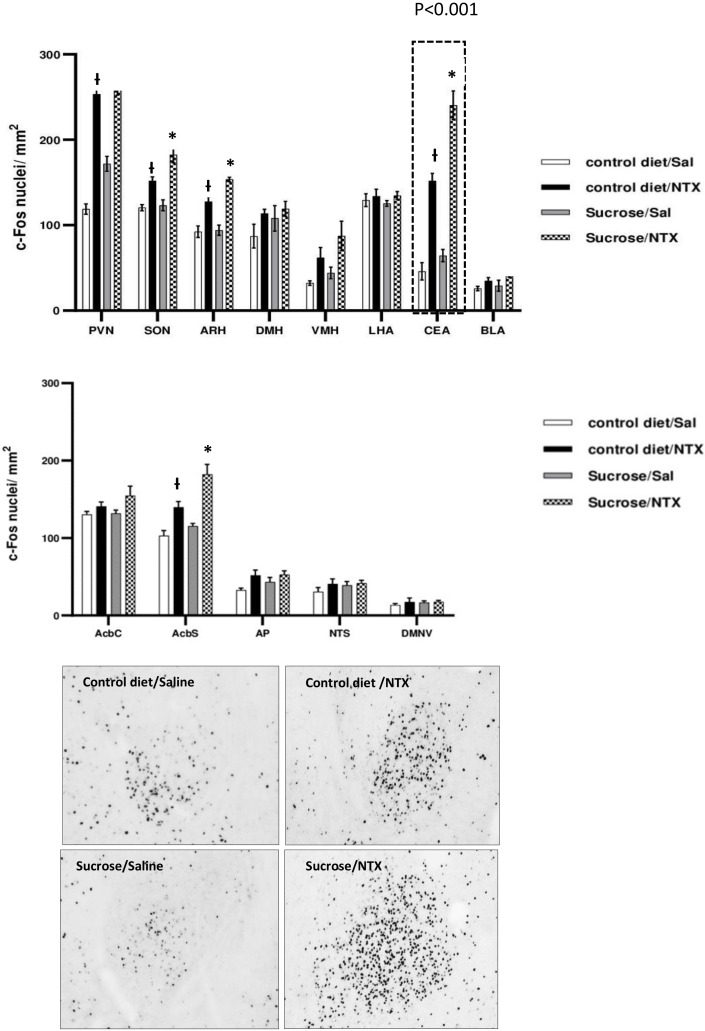
The effect of saline vs NTX (1 mg/kg) on c-Fos immunoreactivity in specific brain sites in control diet vs sucrose consuming rats. * Significantly different from control diet/NTX group. **Ɨ** Significantly different from control diet/saline group. The photomicrographs depict c-Fos immunoreactivity in the central nucleus of the amygdala of rats exposed to standard vs sucrose-enriched diet that received a saline or NTX injection. The CeA was the site where the highest level of drug x diet interaction of all regions examined in our study was noted.

Per the two-way ANOVA analysis, the effect of diet was significant in the PVN (*p* = 0.0021, F_(1,16)_ = 13.45), SON (*p* ≤ 0.0193, F_(1,16)_ = 6.759), ARC (*p* ≤ 0.0233, F_(1,16)_ = 6.294), DMH (*p* ≤ 0.0196, F_(1,16)_ = 6.728), VMH (*p* = 0.0017, F_(1,16)_ = 14.25), CEA (*p* < 0.0001, F_(1,16)_ = 111.7), BLA (*p* = 0.0094, F_(1,16)_ = 8.713), and AcbS (*p* < 0.0001, F_(1,16)_ = 8.524). Finally, there was a statistically significant drug–diet interaction in the PVN (*p*= 0.0289, F_(1,16)_ = 5.760), SON (*p* = 0.0443, F_(1,16)_ = 4.762), ARC (*p* = 0.0444, F_(1,16)_ = 4.759), AcbS (*p* = 0.0231, F_(1,16)_ = 6.308), and—with the most statistically significant difference—the CEA (*p* < 0.0001, F_(1,16_) = 47.71) (Figure 7). In addition, a *t*-test analysis revealed that c-Fos-IR to be more significantly elevated in the control diet/NTX groups in the PVN, SON, ARC, CEA, and AcbS than control diet/saline groups (*p* < 0.001 for all groups). c-Fos IR in the sucrose/NTX groups in the PVN (*p* < 0.0001), SON (*p* = 0.0008), ARC (*p* < 0.0001), CEA (*p* < 0.0001), and AcbS (*p* = 0.0008) was higher than in the sucrose/saline group. Moreover, we found c-Fos-IR to be significantly elevated in the sucrose/NTX groups in the SON (*p* = 0.0188), ARC (*p* = 0.0033), CEA (*p* < 0.0001), and AcbS (*p* = 0.0213) compared to the control diet/NTX group (Figure 7).

Marc-Fos immunoreactivity was not related to overall daily calorie intake over the course of the study: On average, rats consumed 85.26+/−8.41 g of sucrose solution per night. This feeding regimen produced similar total daily energy intake per day per animal of 90.42+/−6.34 kcal (control diet cohort) and 96.14+/−5.23 kcal (palatable sucrose diet cohort), and average body weight did not differ between the cohorts (609.41+/−16.5 g, control; 621.72+/−26.2 g, palatable sucrose). Since on the experimental day immediately after the saline or NTX injection, all tastants were removed from the cage, the c-Fos data reflect only the action of the drug without the interference of different amounts of ingested food or liquid.

## 4. Discussion

Sugar-rich diets are readily consumed by humans and laboratory animals independent of actual energy needs of the organism. The idea that sugars can be reinforcing and even addictive has been popularized by the media and some non- and for-profit organizations and has received support from the scientific community [25,26,35]. Various neural circuits have been identified that are involved in the reinforcing qualities of sugars. These include the mesolimbic dopamine system as well as endogenous opioid [25,26,35]. In the current study, we asked whether sucrose could mimic the effect of morphine on naltrexone discrimination in rats.

Rats naïve to exogenous opioid receptor agonists cannot be trained to discriminate the opioid antagonists naloxone or naltrexone except at extraordinarily large doses [32,33,34]. Such discrimination trials suggest that blockade of endogenous opioid binding to opioid receptors does not result in a perceivable interoception. However, if rats are given morphine prior to training for naltrexone, they can be trained to press the appropriate naltrexone-associated lever [32,33,34]. In the current study, we found that chronic ingestion of a sucrose solution allowed rats to discriminate naltrexone using a standard operant drug-discrimination protocol. This suggests that sucrose can mimic the effects of morphine in such a model perhaps by inducing the release of endogenous opioids or affecting opioid receptors and thereby opioid tone that can be inhibited by NTX. This finding is supported by other research. For example, Jewett et al. [31] found that chronic sucrose ingestion significantly enhanced the ability of rats to discriminate doses nalbuphine by three-fold. Nalbuphine produces discriminative stimulus effects as a low-efficacy, partial agonist at mu-opioid receptors in Sprague–Dawley Rats [36]. The discrimination studies we conducted above expand upon the various opioid-mediated behavioral effects that can be altered by chronic sucrose consumption.

The increase in opioid function allows the opioid-antagonist naltrexone to serve as a discriminative stimulus at doses associated with opioid antagonism. Naltrexone (3.2 mg/kg) represents the largest naltrexone dose in most relevant literature [37,38,39]. We examined the ability of different naltrexone doses to serve as a discriminative stimulus. All subjects learned to discriminate 0.32–3.2 mg/kg naltrexone, and a minority of subjects learned to discriminate 0.1 mg/kg NTX from saline. The subtype(s) of opioid receptors that may contribute to the discriminative stimulus effects of naltrexone in this paradigm is currently unclear.

The increase in opioid function that allows naltrexone to serve as a discriminative stimulus is not dependent on the concentration of sucrose consumed. The acquisition of naltrexone as a discriminative stimulus was not different between subjects drinking 25% sucrose or subjects drinking 32% sucrose. Sucrose intake (g/day) was similar between the two groups (data not shown).

The acquisition and maintenance of the discrimination is related to longer-term changes in opioid function. We determined that acute substitution of water for sucrose (given in the hour before the discrimination session) had no impact on naltrexone’s ability to serve as a discriminative stimulus. That indicates that the changes to the opioid system are relatively long lasting.

We determined that chronic water substitution (2 weeks) results in a decrease or resetting of the opioid system such that naltrexone can no longer serve as a discriminative stimulus. This finding complements the lack of effect of acute water substitution. Reinstating chronic, intermittent sucrose consumption resulted in fairly rapid reacquisition of naltrexone as a discriminative stimulus. As one may predict, the time to reacquire the discriminative stimulus was considerably less than the time to initially learn to discriminate between naltrexone and saline.

In the current study, we also evaluated the effect of naltrexone versus saline injection on c-Fos immunoreactivity in a variety of brain sites involved in feeding behavior in rats drinking a 25% sucrose solution or water in a chronic intermittent exposure paradigm. In order to mimic the discrimination studies, the rats were given not only the sucrose for 10 weeks, but they also received random daily injections of either saline or 1 mg/kg naltrexone throughout this time period (thus, they were familiar with interoceptive effects of both injectants). On the final day, rats were injected with naltrexone or saline. We found that the NTX increases c-Fos IR in selected sites, but we found that the magnitude of the change in c-Fos IR was higher in the sucrose/NTX group than in the sucrose/saline group in a variety of brain sites. Thus, in line with many reports suggesting that habitual sugar intake elevates opioid tone in the brain (for review, see, e.g., [40]), in the current experiments, we found that sucrose ingestion enhances the effect of blockade of opioid receptors with NTX on c-Fos IR in a variety of brain nuclei. Among all the sites where a positive drug–diet interaction was found, the CeA was most highly significant (* *p* < 0.0001). These results are similar to those found by Pomonis et al. [28], who studied the effects of three weeks of consumption of a 10% sucrose solution on naloxone-induced changes in c-Fos IR in rat brains. They found the greatest effect in the central nucleus of the amygdala and a significant interaction in the periaqueductal gray. In the current study, we found more brain sites that had significant drug–diet interactions reflected in c-Fos IR levels; however, it should be noted that in our study, the drug was different (naltrexone rather than naloxone).

Furthermore, it should be emphasized that the sucrose exposure in our experiment was limited to the night time. This is a stark contrast with the Pomonis’ setup, in which the sugar solution was available at all times, completely abolishing the anticipation of exposure to the palatable diet and making the sweet ingestant into a more “standard” (albeit still palatable and rewarding) food. Nonetheless, while it is intuitive that the CEA or AcbS, areas critical for emotional processing of food reward, showed a significant change in activation, the scope of changes in hypothalamic regions typically associated with homeostatic control of energy balance is striking. For example, activation of the PVN and SON, areas where oxytocin and vasopressin neurons are amassed, convey termination of feeding, and historically, they have been viewed as critical mediators of “fullness” (understood as a combination of stomach distension and changes in plasma osmolality and circulating nutrient/hormone levels) [41,42]. Similarly, the ARC where melanocortin, Agouti-related protein, and neuropeptide Y-synthesizing cells are localized is part of the circuit that ensures proper energy balance [42,43]. Yet, mapping of opioid receptors shows that they are abundantly expressed also throughout the “homeostatic” network of sites [44]. This interrelationship between the palatability of a diet and pharmacological blockade of opioid receptors serves as yet another indicator of a functional intersect between eating for pleasure and eating for calories. It exemplifies how exposure to rewarding tastants can affect also those brain areas that are involved in the regulation of the hunger-satiation processes, possibly shifting the dynamic balance between the mechanisms that affect energy control [40]. Furthermore, it suggests that drugs, such as naltrexone, and thus molecules that target predominantly eating driven by palatability, produce a profound response in the homeostatic brain circuit, thereby being able to shift reward processing of consumption along with the perceived need for ingesting calories.

The results of the current study reinforce the notion that sucrose/sugars can affect opioid circuits. Both drug discrimination and c-Fos IR were impacted by sucrose imbibition. More than a decade ago, Hoebel’s laboratory [26,45,46,47] found that chronic ingestion of sugars can result in naloxone/naltrexone-induced withdrawal effects similar to those seen after morphine administration. These include central levels of acetylcholine and dopamine sampled by micro-dialysis and behaviors such as teeth chattering and head shakes.

In conclusion, we found that imbibition of sucrose can alter the ability of rats to discriminate naltrexone from saline injections. In addition, both naltrexone and sucrose have significant interactive effects on activation, as reflected by c-Fos IR, of brain regions known to be involved in feeding behavior.

## Data Availability

Data presented in this study will be made available by the corresponding author upon request.
